# Isoniazid-induced subacute cutaneous lupus erythematosus in an HIV-positive woman: a rare side effect to be aware of with the current expansion of isoniazid preventive therapy

**DOI:** 10.11604/pamj.2018.29.200.12081

**Published:** 2018-04-06

**Authors:** Lameck Bonaventure Luwanda, Anna Gamell

**Affiliations:** 1 Ifakara Health Institute, Ifakara, United Republic of Tanzania; 2 Swiss Tropical and Public Health Institute, Basel, Switzerland; 3 University of Basel, Basel, Switzerland

**Keywords:** Tuberculosis, HIV, isoniazid preventive therapy

## Image in medicine

A 40-year-old HIV-positive female presented with an erythematous macular eruption involving the malar and periorbital area, the forehead and neck of six weeks of duration (A). She had initiated antiretroviral treatment with co-formulated tenofovir/lamivudine/efavirenz QD plus isoniazid preventive therapy (IPT) 18 weeks before with CD4 counts of 496 cells/μl (25%). Drug-induced subacute cutaneous lupus erythematous (DI-SCLE) secondary to isoniazid was clinically diagnosed and isoniazid was stopped. Antinuclear antibody (ANA), anti-SSA/Ro, anti-SSB/La, anti-Sm, anti-RNP and antihistone antibodies were negative. Complete blood count, eGFR and liver transaminases were normal. Three months after stopping isoniazid, the skin lesions resolved completely, supporting the diagnosis of isoniazid-induced SCLE (B). DI-SCLE with negative ANA has been described, but there are no reports in HIV-infected patients. With the current expanded provision of IPT to people living with HIV, it is important to be aware of this rare side effect of isoniazid despite the negativity of antibody assays.

**Figure 1:**
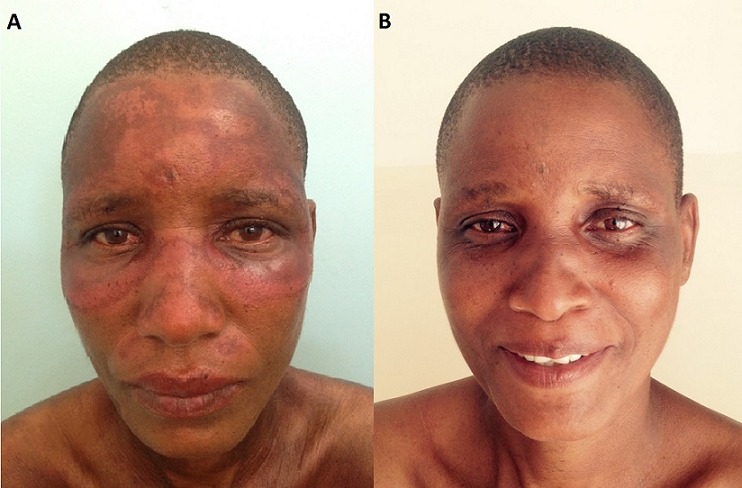
(A) malar, periorbital and forehead macular eruption after starting antiretroviral drugs and isoniazid preventive therapy; (B) a clear skin three months after stopping isoniazid

